# Supplementary material for: Testicular Caspase-3 and β-Catenin Regulators Predicted via Comparative Metabolomics and Docking Studies
Source: Metabolites. 2020 Jan 11;10(1):31. doi: 10.3390/metabo10010031 (PMC7022381; doi:10.3390/metabo10010031)
Supplement: Supplementary file 1 [file metabolites-10-00031-s001.pdf]

Supplementary materials for Metabolites-MDPI

Supplementary Table S1: LC-HRESIMS analysis of *Apium graveolens* extract

**Accurate *m/z***	**Quasi-form**	**Suggested formula**[a]	**Tentative identification**[b]
163.0385	$[M+H]^+$	C9H6O3	7-hydroxy-2H-chromen-2-one
193.1227	$[M+H]^+$	C12H16O2	Senkyunolide A
217.0499	$[M+H]^+$	C12H8O4	Bergapten
247.0605	$[M+H]^+$	C13H10O5	isopimpinellin
261.1852	$[M+H]^+$	C17H24O2	Falcarindiol
301.0710	$[M+H]^+$	C16H12O6	Chrysoeriol
303.0495	$[M+H]^+$	C15H10O7	Quercetol
333.1183	$[M+H]^+$	C14H20O9	Leonuriside
373.1491	$[M+H]^+$	C17H24O9	Syringin
387.2015	$[M+H]^+$	C19H30O8	citroside A or citroside B
401.3773	$[M+H]^+$	C28H48O	Campesterol
413.3775	$[M+H]^+$	C29H48O	Stigmasterol
433.1125	$[M+H]^+$	C21H20O10	Apigenin 7-O-glucoside
449.1075	$[M+H]^+$	C21H20O11	Luteolin 7-O-glucoside
565.1554	$[M+H]^+$	C26H28O14	apiin
663.6077	$[M+H]^+$	C46H78O2	Campesteryl linoleate
675.6073	$[M+H]^+$	C47H78O2	Stigmasteryl linoleate

[a] High Resolution Electrospray Ionization Mass Spectrometry (HRESIMS) using XCalibur 3.0 and allowing for M+H / M+Na adduct.
[b] The suggested compound according to Dictionary of Natural Products (DNP 23.1, 2015 on DVD) and Reaxys online database.

Supplementary Table S2: LC-HRESIMS analysis of *Anethum graveolens* extract

**Accurate *m/z***	**Quasi-form**	**Suggested formula**[a]	**Tentative identification**[b]
163.0383	$[M+H]^+$	C9H6O3	7-hydroxy-2H-chromen-2-one
163.0755	$[M+H]^+$	C10H10O2	Safrole
179.0335	$[M+H]^+$	C9H6O4	Aesculetin
217.0496	$[M+H]^+$	C12H8O4	Bergapten
223.0963	$[M+H]^+$	C12H14O4	Dillapiole
287.1123	$[M+H]^+$	C13H18O7	Gastrodin
333.1909	$[M+H]^+$	C16H28O7	*p*-menth-2-ene-diol β-D-glucoside
351.2015	$[M+H]^+$	C16H30O8	*p*-menthane triol β-D-glucoside
355.1025	$[M+H]^+$	C16H18O9	Chlorogenic acid
373.1493	$[M+H]^+$	C17H24O9	Syringin
413.3774	$[M+H]^+$	C29H48O	Stigmasterol
415.3935	$[M+H]^+$	C29H50O	β-sitosterol
595.1655	$[M+H]^+$	C27H30O15	Vicenin

[a] High Resolution Electrospray Ionization Mass Spectrometry (HRESIMS) using XCalibur 3.0 and allowing for M+H / M+Na adduct.
[b] The suggested compound according to Dictionary of Natural Products (DNP 23.1, 2015 on DVD) and Reaxys online database.

Supplementary Table S3: LC-HRESIMS analysis of *Albizia lebbeck* extract

**Accurate *m/z***	**Quasi-form**	**Suggested formula**[a]	**Tentative identification**[b]
183.0915	$[M+H]^+$	C12H10N2	Harmane
243.0873	$[M+H]^+$	C12H10N4O2	Lumichrome
257.0805	$[M+H]^+$	C15H12O4	Isoliquirtigenin
287.0553	$[M+H]^+$	C15H10O6	kaempferol
301.0705	$[M+H]^+$	C16H12O6	Chrysoeriol
305.0655	$[M+H]^+$	C15H12O7	Taxifolin
317.0653	$[M+H]^+$	C16H12O7	Rhamnetin
339.1075	$[M+H]^+$	C16H18O8	*p*-coumaroylquinic acid
407.1850	$[M+H]^+$	C25H26O5	Lupinifolin
413.3776	$[M+H]^+$	C29H48O	Stigmasterol
415.3933	$[M+H]^+$	C29H50O	β-sitosterol
427.3935	$[M+H]^+$	C30H50O	Lupeol
449.1075	$[M+H]^+$	C21H20O11	Quercitrin
485.3110	$[M+H]^+$	C26H44O8	Mollisside A
539.0975	$[M+H]^+$	C30H18O10	Amentoflavone
575.4305	$[M+H]^+$	C35H58O6	Stigmasterol-3-O-β-glucoside
595.1444	$[M+H]^+$	C30H26O13	*p*-coumaroylquercitrin
625.1550	$[M+H]^+$	C31H28O14	Feruloylquercitrin
629.1710	$[M+H]^+$	C27H32O17	Albizinin
765.4422	$[M+H]^+$	C41H64O13	Concinnoside A
781.4730	$[M+H]^+$	C42H68O13	Acutoside A
792.4895	$[M+H]^+$	C43H69NO12	Albiziabioside A
811.1296	$[M+H]^+$	C44H26O16	Albiproflavone
883.5055	$[M+H]^+$	C46H74O16	Pitheduloside C
895.5053	$[M+H]^+$	C47H74O16	Prosapogenin-3
897.4844	$[M+H]^+$	C46H72O17	Albiziasaponin A
911.4995	$[M+H]^+$	C47H74O17	Julibroside A2
924.5313	$[M+H]^+$	C48H77NO16	Albiziatrioside A √
927.4945	$[M+H]^+$	C47H74O18	Albiziasaponin B
952.5261	$[M+H]^+$	C49H77NO17	Julibroside A3
1059.5371	$[M+H]^+$	C52H82O22	Albiziasaponin C

[a] High Resolution Electrospray Ionization Mass Spectrometry (HRESIMS) using XCalibur 3.0 and allowing for M+H / M+Na adduct.
[b] The suggested compound according to Dictionary of Natural Products (DNP 23.1, 2015 on DVD) and Reaxys online database.

Supplementary Table S4: LC-HRESIMS analysis of *Mentha piperita* extract

**Accurate *m/z***	**Quasi-form**	**Suggested formula**[a]	**Tentative identification**[b]
165.0913	$[M+H]^+$	C10H12O2	Eugenol
167.1063	$[M+H]^+$	C10H14O2	Mintlactone
199.1691	$[M+H]^+$	C12H22O2	L-menthyl acetate
221.1902	$[M+H]^+$	C15H24O	β-Betulenol
271.0600	$[M+H]^+$	C15H10O5	5,7-dihydroxy-2-(4-hydroxyphenyl)-4H-1-benzopyran-4-one
301.0705	$[M+H]^+$	C16H12O6	7-O-methyl-seutellarein

315.0861	$[M+H]^+$	C17H14O6	Ladanein
331.0815	$[M+H]^+$	C17H14O7	Thymusin
345.0965	$[M+H]^+$	C18H16O7	Nevadensin
361.0916	$[M+H]^+$	C18H16O8	Rosmarinic acid
487.1445	$[M+H]^+$	C21H26O13	5,7-dihydroxycromone-7-O-rutinoside
579.1709	$[M+H]^+$	C27H30O14	Apigenin 7-O-rutinoside
581.1863	$[M+H]^+$	C27H32O14	Naringenin 7-O-rutinoside
609.1812	$[M+H]^+$	C28H32O15	Diosmetin 7-O-rutinoside

[a] High Resolution Electrospray Ionization Mass Spectrometry (HRESIMS) using XCalibur 3.0 and allowing for M+H / M+Na adduct.
[b] The suggested compound according to Dictionary of Natural Products (DNP 23.1, 2015 on DVD) and Reaxys online database.

Supplementary Table S5: LC-HRESIMS analysis of *Lactuca sativa* extract

**Accurate *m/z***	**Quasi-form**	**Suggested formula**[a]	**Tentative identification**[b]
181.0497	$[M+H]^+$	C9H8O4	Caffeic acid
241.0861	$[M+H]^+$	C15H12O3	Lettucenin A
243.1018	$[M+H]^+$	C15H14O3	Lettucenin B
267.1590	$[M+H]^+$	C15H22O4	9β-hydroxyl-tetrahydrozaluzanin C
277.1070	$[M+H]^+$	C15H16O5	Lactucin
303.0451	$[M+H]^+$	C15H10O7	Quercetol
355.1022	$[M+H]^+$	C16H18O9	Chlorogenic acid
411.1435	$[M+H]^+$	C23H22O7	Lactupicrin
415.3933	$[M+H]^+$	C29H50O	β-sitosterol
427.1960	$[M+H]^+$	C21H30O9	Lactuside A
427.3931	$[M+H]^+$	C30H50O	α-amyrin
463.0870	$[M+H]^+$	C21H18O12	Luteolin-7-O-β-glucuronopyranoside
465.1025	$[M+H]^+$	C21H20O12	Quercetin-3-O- β-glucoside
577.4465	$[M+H]^+$	C35H60O6	Daucosterol

[a] High Resolution Electrospray Ionization Mass Spectrometry (HRESIMS) using XCalibur 3.0 and allowing for M+H / M+Na adduct.
[b] The suggested compound according to Dictionary of Natural Products (DNP 23.1, 2015 on DVD) and Reaxys online database.

Supplementary Table S6: LC-HRESIMS analysis of *Anagallis arvensis* extract

**Accurate *m/z***	**Quasi-form**	**Suggested formula**[a]	**Tentative identification**[b]
287.0553	$[M+H]^+$	C15H10O6	Kaempferol
475.3780	$[M+H]^+$	C30H50O4	Tetrahydroxyolean-12-ene
517.3163	$[M+H]^+$	C30H44O7	Cucurbitacin L
557.3110	$[M+H]^+$	C32H44O8	Cucurbitacin E
611.1609	$[M+H]^+$	C27H30O16	Rutin
721.3792	$[M+H]^+$	C38H56O13	Arvenin I
901.5159	$[M+H]^+$	C46H76O17	Anagallosaponin VI √
943.5265	$[M+H]^+$	C48H78O18	Apoanagallosaponin III ??
1063.5681	$[M+H]^+$	C52H86O22	Anagallisin C √
1105.5785	$[M+H]^+$	C54H88O23	Anagallosaponin V ??
1121.5735	$[M+H]^+$	C54H88O24	Anagallosaponin VIII
1137.5685	$[M+H]^+$	C54H88O25	Anagallosaponin II √
1225.6210	$[M+H]^+$	C58H96O27	Anagallisin A ??
1241.6165	$[M+H]^+$	C58H96O28	Anagallosaponin I ??

1283.6263	$[M+H]^+$	C60H98O29	Anagallosaponin IX

[a] High Resolution Electrospray Ionization Mass Spectrometry (HRESIMS) using XCalibur 3.0 and allowing for M+H / M+Na adduct.
[b] The suggested compound according to Dictionary of Natural Products (DNP 23.1, 2015 on DVD) and Reaxys online database.

Supplementary Table S7: LC-HRESIMS analysis of *Hibiscus sabdariffa* extract

Accurate *m/z*	Quasi-form	Suggested formula[a]	Tentative identification[b]
177.0393	$[M+H]^+$	C6H8O6	Ascorbic acid
191.0184	$[M+H]^+$	C6H6O7	Hibiscus acid
205.0341	$[M+H]^+$	C7H8O7	Hibiscus acid 6-methyl ester
209.0295	$[M+H]^+$	C6H8O8	Hydroxycitric acid
319.0447	$[M+H]^+$	C15H10O8	Gossypetin
335.0397	$[M+H]^+$	C15H10O9	Hibiscetin
413.3775	$[M+H]^+$	C29H48O	Stigmasterol
415.3934	$[M+H]^+$	C29H50O	β-sitosterol
481.0975	$[M+H]^+$	C21H20O13	Gossypetin 7-O-glucoside
577.4460	$[M+H]^+$	C35H60O6	β-Sitosteryl-β-D-galactoside
581.1500	$[M]^+$	$C26H29O15^+$	Cyanidin-3-O-sambubioside
598.1451	$[M]^+$	$C26H29O16^+$	Delphinidin-3-O-sambubioside

[a] High Resolution Electrospray Ionization Mass Spectrometry (HRESIMS) using XCalibur 3.0 and allowing for M+H / M+Na adduct.
[b] The suggested compound according to Dictionary of Natural Products (DNP 23.1, 2015 on DVD) and Reaxys online database.

Supplementary Table S8: LC-HRESIMS analysis of *Calendula officinalis* extract

Accurate *m/z*	Quasi-form	Suggested formula[a]	Tentative identification[b]
317.0655	$[M+H]^+$	C16H12O7	Isorhamnetin
331.1022	$[M+H]^+$	C14H18O9	Irisdichototin F
355.1021	$[M+H]^+$	C16H18O9	Chlorogenic acid
427.3933	$[M+H]^+$	C30H50O	α-amyrin
443.3880	$[M+H]^+$	C30H50O2	Calenduladiol
449.1074	$[M+H]^+$	C21H20O11	Quercetin 3-O-α-L-rhamnoside
457.3673	$[M+H]^+$	C30H48O3	Oleanolic acid
463.1233	$[M+H]^+$	C22H22O11	Isorhamnetin 3-O-α-Lrhamnoside
465.1025	$[M+H]^+$	C21H20O12	Quercetin 3-O-β-D-glucoside
507.1131	$[M+H]^+$	C23H22O13	Quercetin-3-O-(6"-acetyl)-β-D-glucoside
517.1340	$[M+H]^+$	C25H24O12	Cynarin
537.4453	$[M+H]^+$	C40H56	β-carotene
545.1650	$[M+H]^+$	C27H28O12	1,5-di-O-feruloylquinic acid
609.1812	$[M+H]^+$	C28H32O15	Calendoflaside
611.1603	$[M+H]^+$	C27H30O16	Calendoflavobioside
781.4731	$[M+H]^+$	C42H68O13	Calenduloside A
795.4522	$[M+H]^+$	C42H66O14	Spinasaponin A
943.5260	$[M+H]^+$	C48H78O18	Calenduloside C
957.5055	$[M+H]^+$	C48H76O19	Calenduloside H
973.5005	$[M+H]^+$	C48H76O20	Calendasaponin B
1105.5788	$[M+H]^+$	C54H88O23	Calenduloside D
1119.5580	$[M+H]^+$	C54H86O24	Calendasaponin A
1135.5530	$[M+H]^+$	C54H86O25	Calendasaponin D

[a] High Resolution Electrospray Ionization Mass Spectrometry (HRESIMS) using XCalibur 3.0 and allowing for M+H / M+Na adduct.
[b] The suggested compound according to Dictionary of Natural Products (DNP 23.1, 2015 on DVD) and Reaxys online database.

Supplementary Table S9: LC-HRESIMS analysis of *Rosmarinus officinalis* extract

**Accurate *m/z***	**Quasi-form**	**Suggested formula[a]**	**Tentative identification[b]**
285.0756	$[M+H]^+$	C16H12O5	Genkwanin
287.0554	$[M+H]^+$	C15H10O6	Scutellarein
301.2160	$[M+H]^+$	C20H28O2	Barbatusol
317.2110	$[M+H]^+$	C20H28O3	Rosmaridiphenol
331.1901	$[M+H]^+$	C20H26O4	Carnosol
333.2062	$[M+H]^+$	C20H28O4	Carnosic acid
346.2011	$[M+H]^+$	C20H27NO4	Rosmaricin
347.0760	$[M+H]^+$	C17H14O8	Rosmarinic acid
359.1850	$[M+H]^+$	C21H26O5	Rosmaquinone A
377.1960	$[M+H]^+$	C21H28O6	14-hydroxy-7-O-methyl rosmanol
443.3881	$[M+H]^+$	C30H50O2	Betulinol
447.1284	$[M+H]^+$	C22H22O10	Acacetin 7-O-β-D-glucoside
449.1077	$[M+H]^+$	C21H20O11	Luteolin 7-O-β-Dglucoside
457.3677	$[M+H]^+$	C30H48O3	Betulinic acid
473.3622	$[M+H]^+$	C30H48O4	Hydroxybetulinic acid
579.1705	$[M+H]^+$	C27H30O14	Apigenin-7-rutinoside
611.1972	$[M+H]^+$	C28H34O15	Hesperidin
655.1655	$[M+H]^+$	C32H30O15	6''-O-(E)-feruloylnepitrin

[a] High Resolution Electrospray Ionization Mass Spectrometry (HRESIMS) using XCalibur 3.0 and allowing for M+H / M+Na adduct.
[b] The suggested compound according to Dictionary of Natural Products (DNP 23.1, 2015 on DVD) and Reaxys online database.

Supplementary Table S10: LC-HRESIMS analysis of *Calotropis procera* extract

**Accurate *m/z***	**Quasi-form**	**Suggested formula[a]**	**Tentative identification[b]**
373.2370	$[M+H]^+$	C23H32O4	Uzarigenone
389.2322	$[M+H]^+$	C23H32O5	Corotoxigenin
413.3774	$[M+H]^+$	C29H48O	Stigmasterol
415.3935	$[M+H]^+$	C29H50O	β-sitosterol
421.3466	$[M+H]^+$	C30H44O	Calotroprocerone A
423.3620	$[M+H]^+$	C30H46O	Calotroprocerol A
425.3776	$[M+H]^+$	C30H48O	Proceroleanenol B
465.3725	$[M+H]^+$	C32H48O2	Calotroproceryl acetate A
519.2951	$[M+H]^+$	C29H42O8	Ischaridin
531.2950	$[M+H]^+$	C30H42O8	Calactin
533.2744	$[M+H]^+$	C29H40O9	Calotropin
547.2900	$[M+H]^+$	C30H42O9	Calotoxin
577.3003	$[M+H]^+$	C31H44O10	Proceraside A
588.2624	$[M+H]^+$	C31H41NO8S	Uscharin
602.2780	$[M+H]^+$	C32H43NO8S	15β-hydroxyuscharin
611.1605	$[M+H]^+$	C27H30O16	Quercetin 3-O-neohesperidoside
765.4424	$[M+H]^+$	C41H64O13	Digitoxin
1189.6517	$[M+H]^+$	C63H96O21	Calotroposide H

1369.6789	$[M+H]^+$	C68H104O28	Calotroposide L
1513.7576	$[M+H]^+$	C75H116O31	Calotroposide N

[a] High Resolution Electrospray Ionization Mass Spectrometry (HRESIMS) using XCalibur 3.0 and allowing for M+H / M+Na adduct.
[b] The suggested compound according to Dictionary of Natural Products (DNP 23.1, 2015 on DVD) and Reaxys online database.

A

B

C

D

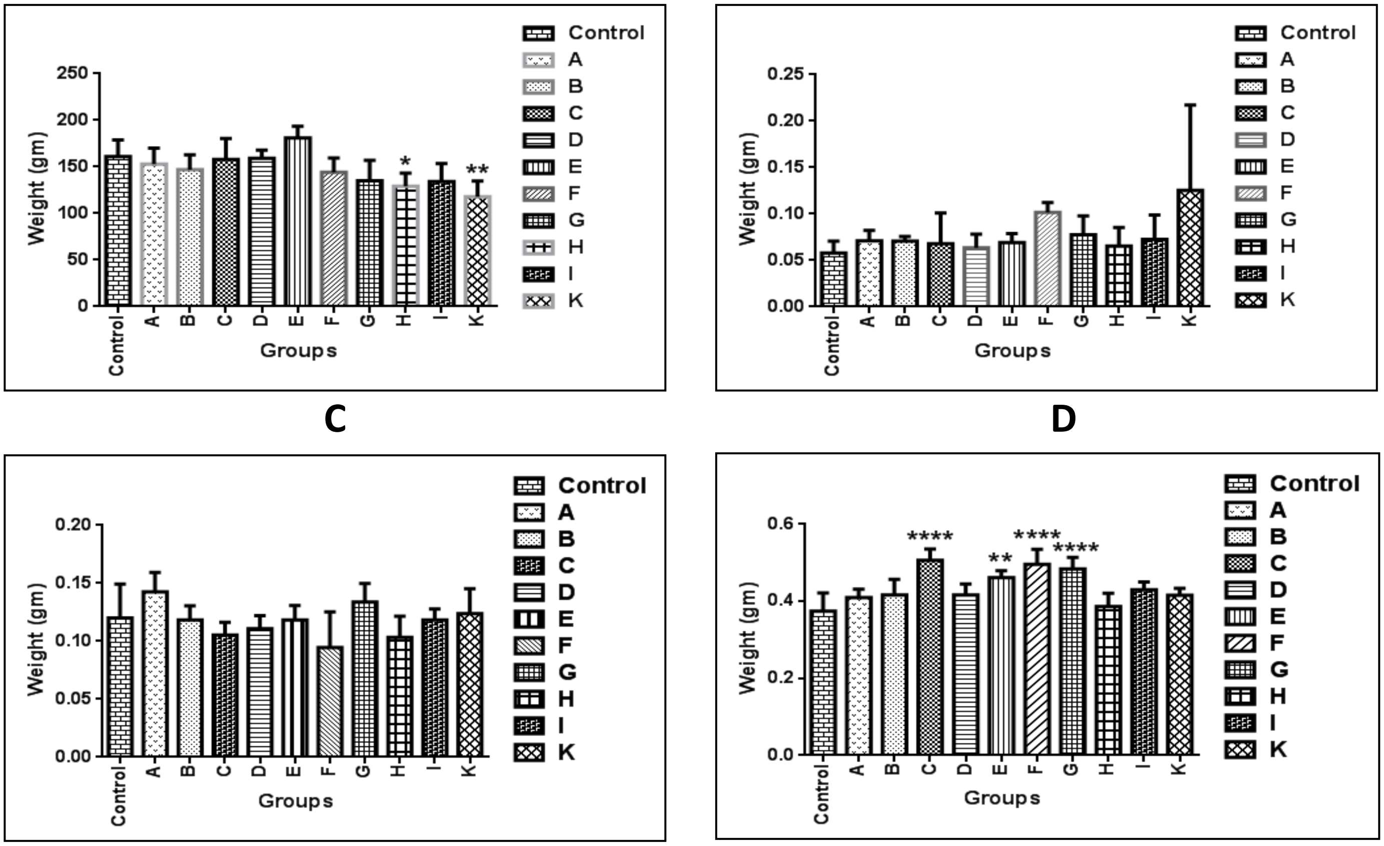

Supplementary Figure. S1. A: Mean body weights of rats exposed to different plant extracts (AG, ANG, AL, MP, LS, AA, CP, RO, CO and HS). **B**: Mean prostate gland relative weights of rats. **C**: Mean seminal vesicle relative weights of rats. **D**: Mean testis relative weight of rats. Values are expressed as mean ± S.E.M. For each group N = 7. * Significantly different from control at $P < 0.05$.

A

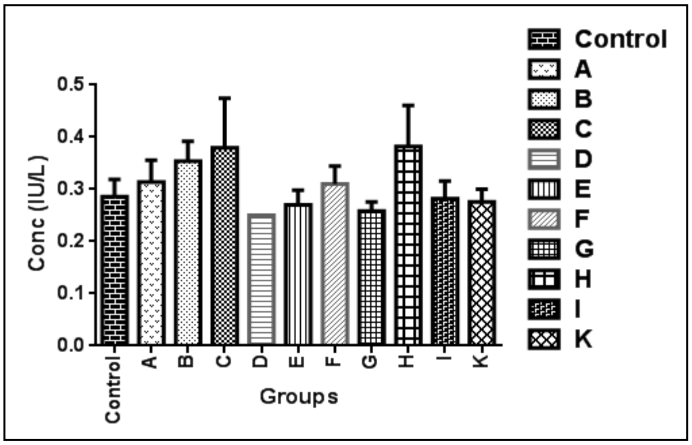

B

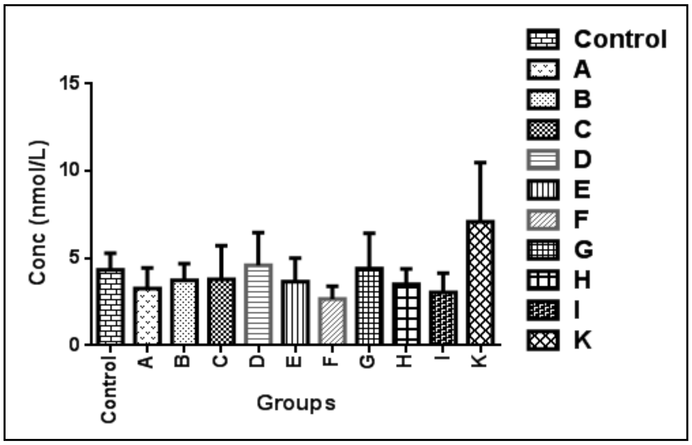

C

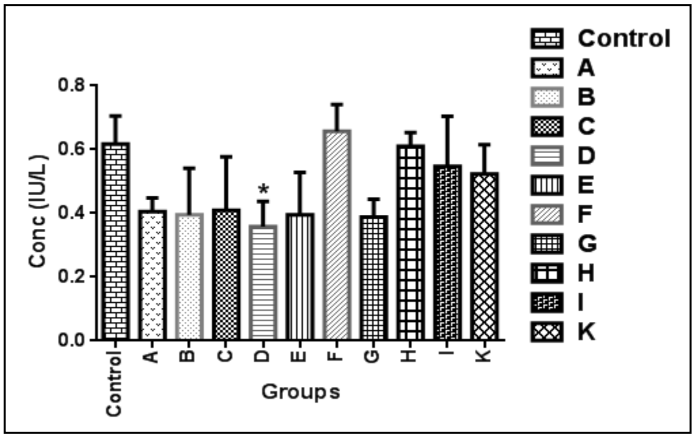

Supplementary Figure S2. Hormonal parameter of treated rats after 2 months of therapy with different plant extracts (AG, ANG, AL, MP, LS, AA, CP, RO, CO and HS). **A**: FSH, **B**: testosterone and **C**: LH.

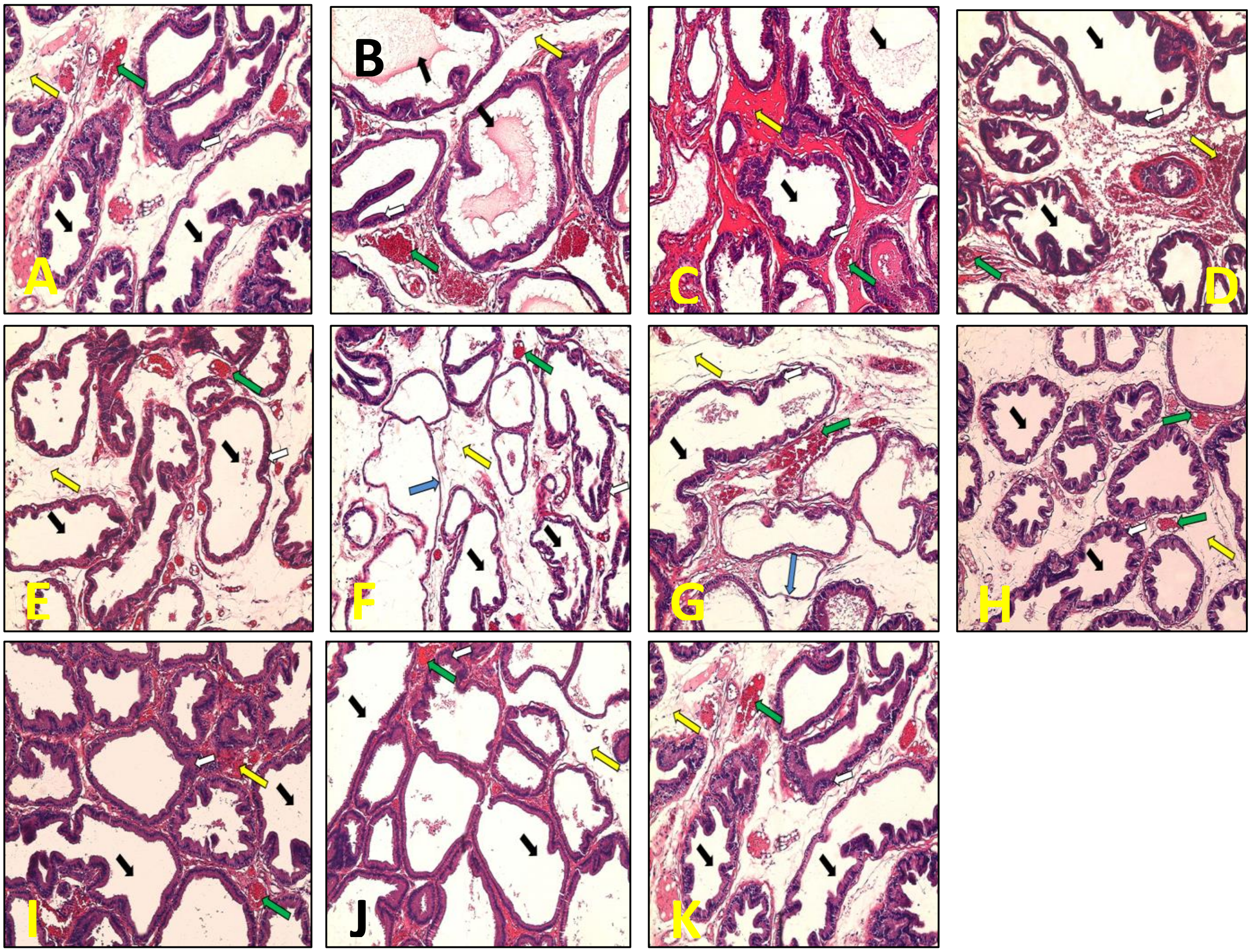

Supplementary Figure S3. A photomicrograph of a paraffin section in seminal vesicle (H&E x100). A. control: The lumen of the glands is highly irregular (black arrow), mucosa is lined with pseudostratified columnar epithelium (white arrow), interstitial hemorrhage (yellow arrow) with congested blood vessels (green arrow). **B. AG**: The same as control. **C. ANG**: The lumen of the glands is highly irregular and stores secretions (black arrow), Mucosa is the same as control, interstitial exudate (yellow arrow) with congested blood vessels (green arrow). **D. AA**: The same as control, **E. MP**: The lumen of the glands and Mucosa are the same as control, interstitial odema (yellow arrow) with congested blood vessels (green arrow). **F. LS**: The lumen of the glands and Mucosa are the same as control, and some glands are lined with flattened epithelium (blue arrow), Interstitial odema (yellow arrow) with congested blood vessels (green arrow). **G. AA**: The same as **LS** group. **H. CC**: The same as **MP** group. **I: RO**: The same as control. **J. CO**: The same as control. **K. HS**: The same as control.

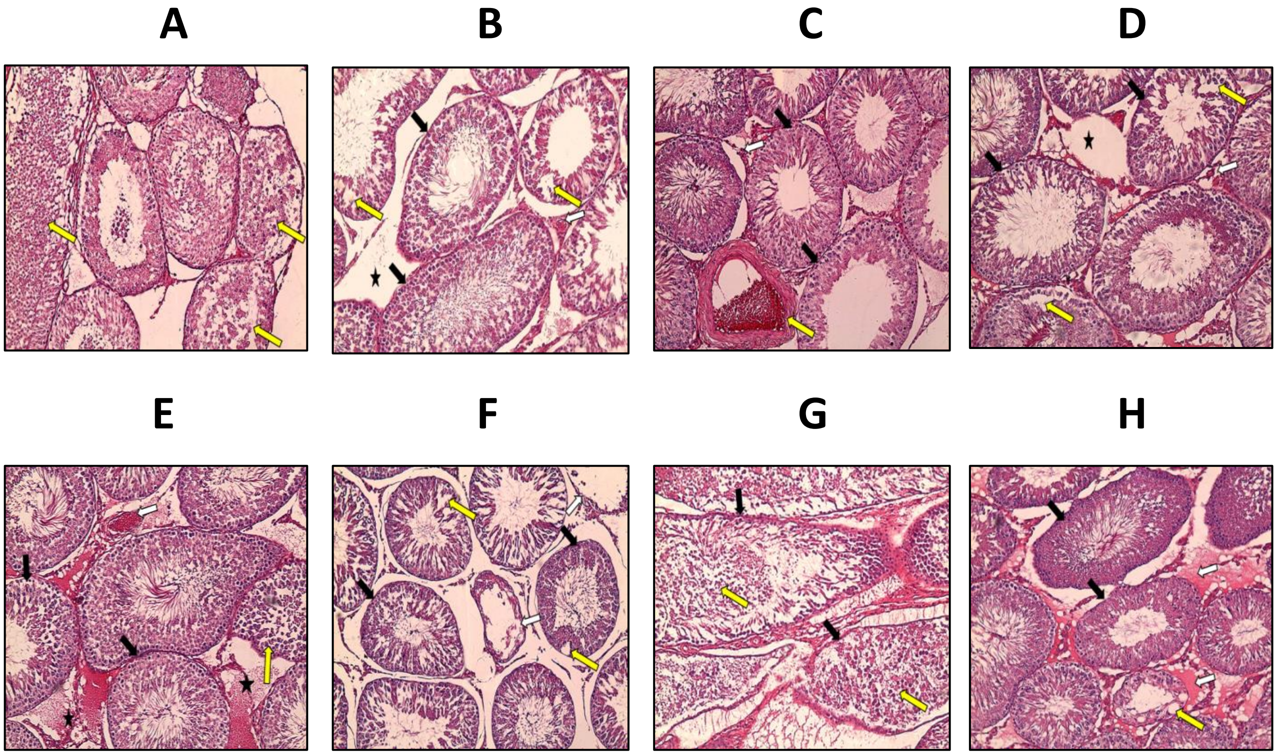

Supplementary Figure S4. **A photomicrograph of a paraffin section in testis (H&E x100) of low significant and non-significant plant extracts. A. Control:** Disorganization of spermatogenic epithelium (yellow arrow). **B. AG**: packed seminiferous tubules (black arrows) lined by stratified germinal epithelium, narrow interstitium containing clusters of interstitial cells and congested blood vessels (white arrows), some areas with wide interstitium (astrix)**,** vacuoles in between spermatogenic epithelium (yellow arrow). **C. ANG**: The same as **AG** group without areas with wide interstitium and vacuoles in between spermatogenic epithelium. **D. MP**: The same as **AG** group. **E. LS**: The same as **AG** group. **F. CC**: Widely separated seminiferous tubules (black arrows) lined by stratified germinal epithelium, vacuoles in between spermatogenic epithelium (yellow arrow), total degeneration of spermatogenic epithelium (white arrow). **G. CO:** Irregular seminiferous tubules (black arrows), disorganization of spermatogenic epithelium (yellow arrow). **H. HS:** Irregular seminiferous tubules (black arrows), wide interstitium with exudate (white arrow), vacuoles in between spermatogenic epithelium (yellow arrow).

Supplementary Table S11: Toxicity and adverse effects doses of investigated plants

Plant name	Toxicity or adverse effects	Reference
***L. sativa* seeds**	The only reported article concerning oral LD_{50} of lettuce was demonstrated by Ghorbani, *et al* on fresh lettuce not seeds which was = 4800 mg/kg in mice (~ 3360 mg/kg in rats*), indicating its high safety.	[1]
	The only adverse effect that reported on *L. sativa* (fresh lettuce) was **potentiating effects on pentobarbital-induced sleep at a dose of 400 mg/kg in mice (~ 280 mg/kg in rat*) but when concomitantly administrated with phenobarbital not itself.**	
	The only reported article concerning Lettuce seeds on male reproductive system utilized 50-200 mg/kg of seeds extract (i.p for 10 days), and no adverse effects, toxicities or mortalities were provided.	[2]
***Apium graveolens* leaves**	The only reported article concerning celery oral LD_{50} was demonstrated by Al-Howiriny, *et al* on aerial parts which was = 7500 mg/kg in rats, indicating its high safety margin	[3,4]
	Acute oral toxicity test revealed no deleterious or toxic symptoms or mortality over a period of 14 days by a dose of 250-500 mg/kg in rats upon investigating studying gastric antiulcer, antisecretory and cytoprotective properties of celery, indicating high safety properties of celery aerial parts.	[4]
	The only reported article concerning *A. graveolens* leaves on male reproductive system utilized 50-150 mg/kg/2 days of leaves extract was injected (IP) for 20 days to rats, and no adverse effects, toxicities or mortalities study were provided.	[4]
***Anethum graveolens* seeds**	The only reported article concerning LD_{50} of dill seeds was demonstrated by Al-Hosseinzadeh, *et al* which was = 3004 mg/kg, i.p., (1500, 6016) and 6098 mg/kg, i.p., (5069, 8056) of aqueous and ethanolic extract, respectively in mice (~2102 mg/kg, and 4269 mg/kg in rats*), indicating its high safety margin when taken orally.	[5]
	It was reported that aqueous and hydroalcoholic extracts of seeds possess male contraceptive effects at doses of 45, 450 and 500, 5000 mg/kg/orally, respectively in rats. And no toxicity study was provided.	[6]
***Calendula officinalis* flowers**	A LD50 of 375 mg/kg and a LD100 of 580 mg/kg has been determined in mice by intravenous and intraperitoneal administration of aqueous extracts. In hydro-alcoholic extracts a LD50 of 45mg/ mouse (sub-cutaneous) and LD50 of 5260 mg/ kg in rats (intravenous) have been reported.	[7]

	In the oral acute study of aqueous extract (2000 mg/kg) in rats, there were no mortality and signs of toxicity. In the subchronic study (250, 1000 mg/kg), several of the blood elements were significantly affected in males and females after 90 days; hemoglobin, erythrocytes, leukocytes and blood clotting time. For blood chemistry parameters, ALT, AST and alkaline phosphatase were affected. Histopathological examination of tissues showed slight abnormalities in hepatic parenchyma that were consistent with biochemical variations observed. These studies indicate that the acute and subchronic toxicities of *C. Officinalis* aqueous extract are low.	[8]
	In the acute toxicity test, hydro-alcoholic extract failed to cause death in the animals after administration of oral doses up to 5000 mg/kg in rats. Oral treatment with hydro-alcoholic extract at 25, 250, 500 and 1000 mg/kg/day for 30 days did not induce hematological alterations. In the biochemical parameters, there was an increase in blood urea nitrogen (BUN) and in alanine transaminase (ALT) levels. Morphological examination of the brain, kidney and heart did not show any alteration.	[9]
	Oral LD_{50} of the ethanolic extract was found to be 2450 mg/Kg in mice (~1715 mg/kg in rats*)	[10]
	It was reported that butanol fraction of flowers possess spermicidal activity, and no toxicity studies concerning male reproductive system were reported.	[11]
***Menthae piperitae* leaves**	Aqueous extract above 5000 mg/kg orally in rats revealed no mortality (LD_{50}), in which the histological changes observed in the selected organs and the biochemical deviation of blood compared to the normal range level were minimal after 14 days (sub-chronic). So, it is suggested to be highly safe.	[12]
	The oral LD_{50} of ethanolic extract of leaves was found to be 3700 mg/kg in rats	[13]
	Aqueous tea was investigated to affect male reproductive system through free administration to rats randomly without fixed daily dose. No toxicity study was provided.	[14]
***Rosmarinus officinalis* leaves**	The median lethal dose (LD_{50}) value of methanolic extract of *Rosmarinus officinalis* leaves was 4125 mg/kg in mice intraperitoneally (~2888 mg/kg in rats*).	[15]
	The oral LD_{50} of rosemary leaves extract has been described as > 8500 mg/kg in rats.	[16]
	The article which discussed the effect of hydroethanolic extract of *Rosmarinus officinalis* leaves has used 500 mg/kg daily fixed dose, and no toxicity study was provided.	[17]
***Calotropis procera* aerial parts**	Oral LD_{50} of ethanolic extract of *C. procera* leaves is 95.52 mg/kg in rats. `	[18]
	C. procera ethanolic extract of leaves could induce marked toxicity in heart and testis with 1/10 or 1/20 LD_{50} (95.52 mg/kg in rats) for a period of 4-8 weeks.	[18]
	200 mg/kg body weight of the aqueous leaves extract was daily administered orally by gavages during 42	[19]

	days in rabbits (~375in rats*). All the rabbits gained weight during the administration period, with an appreciable gain for smaller animals. Significant decrease of ALT and RBC were noticed in the youngest rabbits. A significant increase of serum creatinine level and lymphocytes were also noticed within the group of the juvenile rabbits. Necropsy revealed lesions in kidney and liver, these lesions were further confirmed by histopathology observations that revealed more pronounced pathology with the youngest animals. Although animals in different test groups show some toxic effects; small animals of eight weeks exhibit more effects with more severe lesions.	
	The aqueous leaf extract (50, 75 mg/kg/orally in rats) for 15 day produced significant increase in packed call volume (PCV) but did not influence coagulation time. The extract also produced hypoproteinaemia reflected as hypoalbuminaemia. Similarly the leaf extract also caused elevation in the activities of aspartate aminotransferase and alanine aminotransferase. Although the extract did not produce lesions in the heart, spleen and liver examined, the increase in liver enzyme activities could be due to early liver damage.	[20]
	C. procera ethanolic extract of leaves could induce marked toxicity in kidney by using 1/10 0r 1/20 LD_{50} (~95.52 mg/kg in rats*) for a period of 4-8 weeks, orally.	[21]
	Oral LD_{50} was estimated (940 mg/kg) in rabbits (~1725 mg/kg in rats*) of fresh leaves aqueous extract. 80, 40 and 20 mg/kg/day of the extract were administered orally during sub-acute toxicity study for 14 days in rabbits (~150, 75, 40 mg/kg in rats*), Statistical analysis of aspartate amino transferase (AST), ala-nine amino transferase (ALT), alkaline phosphatase (ALP), albumin and protein showed no significant changes. Changes in packed cell volume (PCV), white blood cells (WBC), haemoglobin (Hb), platelets, and differential leucocyte count (lymphocytes, monocytes, eosinophils, heterophils/neutrophils and basophils) were equally statistically insignificant. However, gross and histopathological examination of some organs and tissues (heart, liver, kidney, brain, small intestine and lungs) revealed lesions.	[22]
	Oral LD_{50} was = 533 mg/kg of leaves ethanolic extract in mice (~373 mg/kg in rats*). Hepato- and renal-toxicities (1/10 or 1/20 LD_{50}) were occurred (~40 or 20 mg/kg in rats*). Also, the oral treatment with the ethanolic crude extract of leaves of *Calotropis procera* at a high dose (1/5 of the LD_{50}) for a prolonged time could inhibit or arrest the spermatogenesis process leading to infertility in male albino mice (~80 mg/kg min rats*).	[23]
	It was observed that the hydroalcholic extract of the leaves of *C. procera* (5000 mg/kg, p.o.) did not induce changes in the behavior of male mice during the first 30 min and for a period of up to 4 h after	[24]

	administration. No death was recorded during the fourteen days of observation. No significant changes in intake of food and water or in body weight were observed throughout the period. The LD*50* could not therefore be estimated and is possibly higher than 5000 mg/kg	
	The oral LD5*0* of the hydroalcoholic extract of stem bark with no signs of acute toxicity at 2000 mg/kg in rats	[25]
	The LD*50* of leaves aqueous extract could not therefore be estimated and is possibly higher than 5000 mg/kg	[26]
	The Oral LD_{50} dose of the flower methanolic extract on mice is found to be 1660 mg/kg	[27]
	Flower aqueous and ethanolic extracts were evaluated for their effect on male reproductive system via using an intraperitoneal dose of 5, 10 mg/30 gm in mice per two days for 20 days. No general toxicity study was provided.	[28]
***Hibiscus sabdariffa* calyces**	The plant extracts are characterized by a very low degree of toxicity. The LD*50* of *H. sabdariffa* calyx extract in rats was found to be above 5000 mg / kg. A single report has suggested that excessive doses for relatively long periods could have a deleterious effect on the testes of rats	[29]
	After 14 days of a single oral administration of aqueous extract (5,000 mg/kg). No signs and differences of the weights or behavior compared to the control rats were observed. An oral administration of aqueous extract at the doses of 50, 100 and 200 mg/kg body weight for 270 days does not cause chronic toxicity in rat.	[30]
	The intraperitoneal LD*50* of the aqueous extract of *Hibiscus sabdariffa* calyx was found to be greater than 5000 mg/kg in mice	[31]
	The LD*50* of rosell calyx aqueous extract was found to be above 5000 mg kg−1 IN rats	[32]
	Hibiscus sabdariffa calyces aqueous extract was investigated to affect male reproductive system by using oral doses of 1100-4600 mg/kg in rats/ day for 60 days. No other toxicological parameters were measured (other body organs).	[33]
	Hibiscus sabdariffa calyces aqueous extract was also investigated to affect the sperm morphology by using 200 mg/kg/orally in mice per day for 30 days. No other toxicological parameters were measured (other body organs).	[34]
Anagallis arvensis	The intraperitoneal LD*50* was 10718 mg/kg of alcoholic extract of *A. arvensis*	[35]
	(1/5 and 1/10 LD*50*) IP [10718 mg/kg.b.wt] for 15 days i.e. 2650 mg/kg causes clinical signs included	[35]

	anorexia, restlessness, diarrhea, thirst, difficult breathing, tremors and ended by coma and death. Hematologically, there were a significant reduction in PCV%, Hb concentration and RBCs count of the intoxicated rats. Concerning kidney function tests, there were a significant increase in urea and creatinine level of the intoxicated rats. Pathologically, the lesions were primarily confined to the urinary system.	
	No reported toxicity studies of *Anagallis arvensis* extracts on male reproductive system. Our article is considered the first article that investigated the effect of this herb on spermatogenesis.	
Albizzia lebbeck pods	The acute oral LD*50* of aqueous extract of stem in bark albino rats of either sex was found to be 2000 mg/kg. LD_0 was found to be 400 g/kg, and LD100 approximately 3200 mg/kg.	[36]
	The intraperitoneal LD*50* of the aqueous methanol extract of seeds of *Albizzia lebbeck* was found to be 82 mg/kg in rats.	[37]
	Oral LD*50* of methanolic extract of *Albizia lebbeck* leaves was considered as 2000 mg/kg in rats.	[38]
	Oral LD*50* of 70% ethanolic extract of *Albizzia lebbeck* bark. Since no mortality was observed at 2000 mg/kg in rats.	[39]
	Oral LD*50* more than 2000 mg/kg without any toxic symptoms in stem bark methanolic extract in mice	[40]
	The LD*50* (mice, oral) is therefore estimated to be beyond 5000 mg/kg body weight of aqueous or alcoholic extract of flowers	[41]
	Oral LD*50* more than 5000 mg/kg without any toxic symptoms in stem bark methanolic extract in mice	[42]
	Its toxicity on male reproductive system was reported when using a fixed daily dose of 200 mg/kg/day/orally of ethanolic extract of pods in rats for 60 days. The RBC and WBC counts, haemoglobin, haematocrit and blood sugar were within the normal range at this dose.	[43]
	The other article that investigated the toxicity of pods on male reproductive system has used 250 mg/kg/orally of triterpene fraction of methanolic extract of pods in rate for 60 days. There were no significant changes in RBC and WBC count, haemoglobin, haematocrit, blood glucose, cholesterol, protein, triglycerides, phospolipids and HDL-cholesterol.	[44]

References

1. Ghorbani, A.; Rakhshandeh, H.; Reza, H. Potentiating Effects of Lactuca sativa on Pentobarbital-Induced Sleep. *Iran. J. Pharm. Res.* **2013**, *12*, 401–406.

2. Ahangarpour, A.; Oroojan, A.A.; Radan, M. Effect of aqueous and hydro-alcoholic extracts of lettuce (Lactuca sativa) seed on testosterone level and spermatogenesis in NMRI mice. *Iran J. Reprod. Med.* **2014**, *12*, 65–72.

3. Fraunfelder, F.W. Ocular side effects from herbal medicines and nutritional supplements. *Am. J. Ophthalmol.* **2004**, *138*, 639–647.

4. Al-Howiriny, T.; Alsheikh, A.; Alqasoumi, S.; Al-Yahya, M.; Eltahir, K.; Rafatullah, S. Gastric antiulcer, antisecretory and cytoprotective properties of celery (Apium graveolens) in rats. *Pharm. Biol.* **2010**, *48*, 786–793.

5. Hosseinzadeh, H.; Karimi, G.; Ameri, M. Effects of Anethum graveolens L. seed extracts on experimental gastric irritation models in mice. *BMC Pharmacol.* **2002**, *2*, 21.

6. Monsefi, M.; Zahmati, M.; Masoudi, M.; Javidnia, K. Effects of Anethum graveolens L. on fertility in male rats. *Eur. J. Contracept. Reprod. Heal. Care* **2011**, *16*, 488–497.

7. Basch, E.; Bent, S.; Foppa, I.; Haskmi, S.; Kroll, D.; Mele, M.; Szapary, P.; Ulbricht, C.; Vora, M.; Yong, S. Marigold (Calendula officinalis L.): An evidence-based systematic review by the natural standard research collaboration. *J. Herb. Pharmacother.* **2006**, *6*, 135–159.

8. Lagarto, A.; Bueno, V.; Guerra, I.; Valdés, O.; Vega, Y.; Torres, L. Acute and subchronic oral toxicities of Calendula officinalis extract in Wistar rats. *Exp. Toxicol. Pathol.* **2011**, *63*, 387–391.

9. Silva, E.J.R.; Gonçalves, E.S.; Aguiar, F.; Evêncio, L.B.; Lyra, M.M.A.; Coelho, M.C.O.C.; Fraga, M. do C.C.A.; Wanderley, A.G. Toxicological studies on hydroalcohol extract of Calendula officinalis L. *Phyther. Res. An Int. J. Devoted to Pharmacol. Toxicol. Eval. Nat. Prod. Deriv.* **2007**, *21*, 332–336.

10. Elzorba, H.; El Banna, H.; Derbala, D. Some Pharmacological & Toxicological Activities of Calendula officinalis Linn. Flower 70% Ethanolic Extract. *Anim. Vet. Sci.* **2016**, *4*, 26.

11. Parkhurst, R.M.; Stolzenberg, S.J. Saponin-Containing Spermatocidal Compositions. *United States Pat.* 1975.

12. Johari, N.Z.; Ismail, I.S.; Sulaiman, M.R.; Abas, F. Acute toxicity and metabolomics analysis of hypocholesterolemic effect of Mentha piperita aqueous extract in Wistar rats. *Int. J. Appl. Res. Nat. Prod.* **2015**, *8*, 1–11.

13. Dhanarasu, S.; Selvam, M.; Al-Shammari, N.K.A. Evaluating the pharmacological

dose (Oral ld50) and antibacterial activity of leaf extracts of Mentha piperita Linn. Grown in Kingdom of Saudi Arabia: a pilot study for nephrotoxicity. *Int J Pharmacol* **2016**, *12*, 195–200.

14. Akdogan, M.; Ozguner, M.; Kocak, A.; Oncu, M.; Cicek, E. Effects of peppermint teas on plasma testosterone, follicle-stimulating hormone, and luteinizing hormone levels and testicular tissue in rats. *Urology* **2004**, *64*, 394–398.

15. El-naggar, S.A.; Abdel-farid, I.B.; Germoush, M.O.; Hassan, A.; Alm-eldeen, A.A.; Abdel-farid, I.B.; Germoush, M.O.; Hassan, A. Efficacy of Rosmarinus officinalis leaves extract against cyclophosphamide-induced hepatotoxicity. *Pharm. Biol.* **2016**, *54*, 2007–2016.

16. Farkhondeh, T.; Samarghandian, S.; Pourbagher-Shahri, A.M. Hypolipidemic effects of Rosmarinus officinalis L. *J. Cell. Physiol.* **2019**, *234*, 14680–14688.

17. Nusier, M.K.; Bataineh, H.N.; Daradkah, H.M. Adverse effects of rosemary (Rosmarinus officinalis L.) on reproductive function in adult male rats. *Exp. Biol. Med.* **2007**, *232*, 809–813.

18. Ahmed, O.M.; Fahim, H.I.; Boules, M.W.; Ahmed, H.Y. Cardiac and testicular toxicity effects of the latex and ethanolic leaf extract of Calotropis procera on male albino rats in comparison to abamectin. *Springer Plus* **2016**, *5*, 1644.

19. B, P.G.; Ahmed, H.; Dawurung, C.; Atiku, A. Influence of age on sub-chronic toxicity of the a queous extract of the leaves of Calotropis procera on rabbits. *J. Toxicol. Environ. Heal. Sci.* **2011**, *3*, 119–126.

20. Ajagbonna, O..; Onifade, K.I.; Suleiman, U. Haematological and Biochemical changes in Rats Given extract of Calotropis procera water. *Sokoto J. Vetrinary Sci.* **1977**, *1*, 36–42.

21. Fahim, H.I.; Ahmed, O.M.; Boules, M.W.; Ahmed, H.Y. Nephrotoxic Effects of Abamectin and Calotropis procera Latex and Leaf Extract in Male Albino Rats. *Am. J. Med. Med. Sci.* **2016**, *6*, 73–86.

22. Mbako, J.D.; Adamu, Z.; Afutu, J.K.; David, S.; Aliyu, A. Toxicity assessment of the aqueous extract of Calotropis procera in rabbits. In Proceedings of the Neurotoxicity and Neurodegeneration: Local Effect and Global Impact--Program and Proceedings of the 13~(th) International Neurotoxicology Association Meeting & 11~(th) International Symposium on Neurobehavioral Methods and Effects in Occupational and Envi; 2011.

23. Toson, E.A.; Habib, S.A.; Saad, E.A.; Harraz, N.H. Toxic and anti-fertility effects of Alocasia macrorrhiza and Calotropis procera ethanolic extracts on male mice. *Int J Biochem Phot.* **2014**, *195*, 328–338.

24. Araújo, A. V; Costa-silva, J.H.; Amorim, E.L.C.; Ferreira, F.; Oliveira, A.F.M. De; Wanderley, A.G. Evaluation of antihyperglycaemic activity of Calotropis procera

leaves extract on streptozotocin-induced diabetes in Wistar rats. *Rev. Bras. Farmacogn. - BrazilianJournal Pharmacogn.* **2013**, *23*, 913–919.

25. Tour, N.S.; Talele, G.S. Gastric antiulcer and antiinflammatory activities of Calotropis procera stem bark. *Brazilian J. Pharmacogn.* **2011**, *21*, 1118–1126.

26. Mohammed, A.; Ibrahim, S.; Bilbis, L. Toxicological investigation of aqueous leaf extract of Calotropis procera (Ait .) R . Br . in Wister albino rats. *African J. Biochem. Res.* **2012**, *6*, 90–97.

27. Mukherjee, B.; Bose, S.; Dutta, S.K. Phytochemical and pharmacological investigation of fresh flower extract of Calotropis procera Linn. *Int. J. Pharm. Sci. Res.* **2010**, *1*, 182–187.

28. Sharma, N.; Jacob, D. Inhibition of fertility and functional alteration in the genital organs of male swiss albino mouse after administration of Calotropis procera flower extract. *Pharm. Biol.* **2001**, *39*, 403–407.

29. Badreldin, H.A.; Naser Al, W.; Gerald, B. Phytochemical, pharmacological and toxicological aspects of Hibiscus sabdariffa L.: a review. *Phyther. Res.* **2005**, *19*, 369–375.

30. Sireeratawong, S.; Itharat, A.; Khonsung, P.; Lertprasertsuke, N.; Jaijoy, K. Toxicity studies of the water extract from the calyces of Hibiscus sabdariffa L. in rats. *African J. Tradit. Complement. Altern. Med. AJTCAM* **2013**, *10*, 122–127.

31. Amos, S.; Binda, L.; Chindo, B.A.; Tseja, A.; Odutola, A.A.; Wambebe, C.; Gamaniel, K. Neuropharmacological effects of Hibiscus sabdariffa aqueous extract. *Pharm. Biol.* **2003**, *41*, 325–329.

32. Onyenekwe, P.C.; Ajani, E.O.; Ameh, D.A.; Gamaniel, K.S. Antihypertensive effect of roselle (Hibiscus sabdariffa) calyx infusion in spontaneously hypertensive rats and a comparison of its toxicity with that in Wistar rats. *Cell Biochem. Funct.* **1999**, *17*, 199–206.

33. Orisakwe, O.E.; Husaini, D.C.; Afonne, O.J. Testicular effects of sub-chronic administration of Hibiscus sabdariffa calyx aqueous extract in rats. *Reprod. Toxicol.* **2004**, *18*, 295–298.

34. Mahmoud, Y.I. Effect of extract of Hibiscus on the ultrastructure of the testis in adult mice. *Acta Histochem.* **2012**, *114*, 342–348.

35. AL-Sultan, S.I.; Hussein, .Y. A.; . A.H. Toxicity of Anagallis arvensis Plant. *Pakistan J. Nutr.* **2003**, *2*, 116–122.

36. Tripathi, R.M.; Sen, P.C.; Das, P.K. Studies on the mechanism of action of Albizzia lebbeck, an Indian indigenous drug used in the treatment of atopic allergy. *J. Ethnopharmacol.* **1979**, *1*, 385–396.

37. Besra, S.E.; Gomes, A.; Chaudhury, L.; Vedasiromoni, J.R.; Ganguly, D.K.

Antidiarrhoeal Activity of Seed Extract of Albizzia lebbeck Benth . *Phyther. Res.* **2002**, *533*, 529–533.

38. Sivakumar, B.; Velmurugan, C.; Kumar, P.L. Diuretic Activity Of Methanolic Extract Of ‘ Albizia lebbeck ’ . *Int. J. PharmTech Res.* **2016**, *5*, 404–406.

39. Patel, T.; Shirode, D.; Roy, S.P.; Kumar, S.; Setty, S.R. Evaluation of Antioxidant and Hepatoprotective effects of 70 % ethanolic bark extract of Albizzia lebbeck in rats. *Int. J. Res. Pharm. Sci* **2010**, *1*, 270–276.

40. Manjulatha, K. Protective effect of bark extracts of Albizzia lebbeck Benth on peptic ulcers induced by physical and chemical agents in rodents.

41. Mahdi, T.S.; Al-azzawie, H.F. Hepatoprotective activity of Albizia Lebbeck flowers extract in CCL4 induced liver toxicity mice. *Iraqi J. Sci.* **2016**, *part A*, 55–63.

42. Kalia, S.; Bagai, U.; Gorki, V. Effect of Albizia lebbeck on liver and kidney function of Plasmodium berghei infected mice. *Int. J. Pharma Sci. Res.* **2015**, *6*, 575–581.

43. Gupta, R.S.; Kachhawa, J.B.S.; Chaudhary, R. Antifertility effects of methanolic pod extract of Albizzia lebbeck (L.) Benth in male rats. *Asian J. Androl.* **2004**, *6*, 155–159.

44. Chaudhary, R.; Gupta, R.S.; Kachhaw, J.B.S.; Singh, D.; Verma, S.K. Inhibition of spermatogenesis by Triterpenes of Albizia lebbeck (L .) Benth pods in male albino rats . *J. Nat. Remedies* **2007**, *7*, 86–93.
